# Human nail usage as a Bio-indicator in contamination monitoring of heavy metals in Dizajabaad, Zanjan province-Iran

**DOI:** 10.1186/s40201-014-0147-x

**Published:** 2014-12-14

**Authors:** Abdolhossein Parizanganeh, Abbasali Zamani, Vahid Bijnavand, Behzad Taghilou

**Affiliations:** Environmental Science Research Laboratory, Department of Environmental Sciences, Faculty of Science, University of Zanjan, Zanjan, 45371-38791 Iran; Department of Environmental Science, Faculty of Science, University of Zanjan, Zanjan, 45371-38791 Iran

**Keywords:** Biomonitoring, Bio-indicator, Dizajabaad village, Finger nail, Heavy metals

## Abstract

**Background:**

Due to pedogeochemical background and anthropogenic sources, heavy metal contamination of soil is a widespread problem in some parts of Zanjan province located in North West Iran. In this study an affected area located near National Iranian Lead and Zinc Company (NILZ) was selected for detailed study.

**Methodology:**

Thirty soil samples and eighteen leaf samples were collected and analyzed for heavy metal contamination by Inductively Coupled Plasma-Optical Emission Spectroscopy (ICP-OES). Both soil and plant samples were found to be basically polluted by Pb (72.60 and 97.11), Zn (546.47 and 166.61), and Cd (1.80 and 1.55) mg. kg^−1^ respectively. High concentrations of these elements in soil and plant species signifies possible health risks to humans. The distribution maps drawn using Arc GIS (10) show high concentrations of these toxic metals around Dizajabaad village. To assess vulnerability and health risks of metal concentrations in human bodies’ twenty nine fingernail samples were also collected from people living in this village.

**Results and discussion:**

Analysis for different heavy metal contents of nail samples reveals very high levels of the same toxic elements (Pb = 15.15, Cd = 1.18, As = 15.47, Zn = 68.46 and Ni = 18.22 mg. kg^−1^) compared with samples collected from an unaffected area and available values indicated in the literature. Further, high concentration of heavy metals in the nail samples suggests long term exposure of inhabitants to these toxic metals.

## Introduction

Heavy metals such as lead (Pb), cadmium (Cd), zinc (Zn) and nickel (Ni) occur naturally in water, soil and biota. Their concentrations depend on local geology, local addition from mining and industry and/or globally distributed pollution [1-5]. Elevated levels of these metals in the environment may arise from anthropogenic sources, including consumption of food from contaminated environments [6-12]. The increasing demand for environmental and food safety has stimulated research regarding the risk associated with environmental exposure and consumption of food contaminated with heavy metals [13]. Assessment of chronic exposure to essential/nonessential elements is an area of emerging interest in environmental and nutritional epidemiology.

Given the presence of multiple exposure pathways, the use of biomarkers lends promise to reduce measurement error in traditional exposure assessments, which often rely on recall or aggregate exposure measurements [14,15]. Biomonitoring of metal concentrations in human bodies have significant importance, as their amounts in biological samples (hair, nails, blood, saliva, and urine) can be utilized as a medium metal contamination index [16]. The simplicity with which hair and nails can be sampled, transported and handled, and generally higher elemental concentrations compared to other biological media, such as blood and urine makes hair and nails to be more suitable and convenient tissues for monitoring localized exposure to toxic metals [17,18].

This paper focuses on the utility of nail clippings in biomonitoring of metal concentrations in human bodies. Nail clippings have advantages over the other biological materials frequently analyzed for heavy metals content as they can be easily collected as against bone samples. Human nails are composed of high-sulfur and hard keratins, the fibrous proteins [19]. Many elements bind to keratin present in fingernails and toenails [20]. The element content of nails tends to vary from one geographical region to another, depending on the natural background conditions [21-24]. The time window of exposure reflected by the nail clipping is dependent on the distance of the clipping and the rate of nail growth. Several known biological factors such as age, gender, and other individual-level characteristics can influence nail growth [25,26]. Although growth rates vary among individuals, fingernails grow at an average of 0.1 mm/day while a normal fingernail takes about 6 months to grow out completely [25]. Since concentration of metals in nails reflects their mean level in human body during a period of 12–18 months [27-31], its use is far from being the universal tool for monitoring long exposures to environmental pollutants [32].

Presence and high concentrations of some heavy metals; notably Pb, Zn, Cd and Ni, in soil & plant samples collected from NILZ Company and neighboring area (Dizajabaad village) were determined with ICP-OES. Previous studies have also reported high levels of these heavy metals in soil, which are likely to accumulate in food grown locally and cause heavy metal risk to consumers [33,34]. Extended data are variability of metals across a contaminated area can predict metal variation in nails. To this purpose, the concentration of five heavy metals (Pb, Cd, Zn, As, and Ni) in the nail samples of the inhabitants in the studied area were analyzed as biomarker of long term heavy metal exposures from contaminated environment. This study was carried out in summer 2012 in the Environmental Science Research Laboratory, University of Zanjan, Zanjan-Iran.

## Materials and methods

### The study area

**Figure 1:**
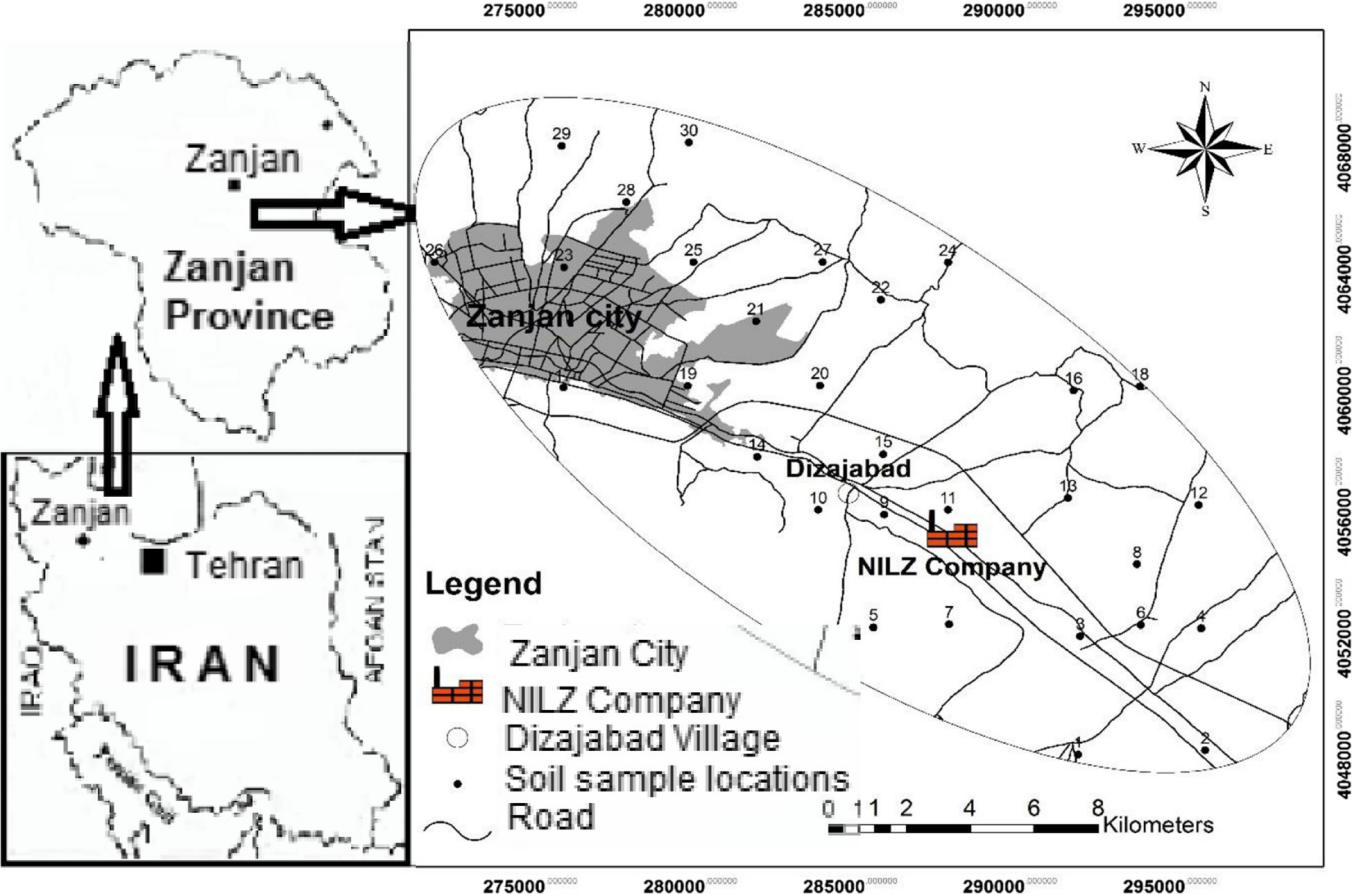
**Location map of the studied area indicating sampling points.**

Valuable reserves of lead and zinc are available in Angoran area in Zanjan province located in northwest Iran. Mining, transportation of concentrated ore by trucks and smelting units within the province presents a risk of contamination of soils, plants, surface/ground water resources and its entrance into the food chain. Many related industries are developed in Zanjan province, notably, NILZ Company which is one of the largest of its kind in the Middle East with a high market capitalization. The Company which plays an important role in the local economy was established in 1994 with a current consumption of about 300,000 tons of raw ore and an annual production of 55000 tons of lead and zinc. The tailing from NILZ, estimated to be about 2.5 million tons, contains a variety of toxic elements, notably Pb, Zn, and Cd [35]. They are damped in the vicinity of the company and exposed to wind and rain contributing to soil, surface and groundwater contaminations. This research was focused on the environmental impacts of NILZ Company on its nearby inhabited areas notably people residing in Dizajabaad village (36° 66′ N, 48°48′ E). The investigated area has a cold and dry climate characterized by cool and dry summers and cold, snowy winters. Figure 1 shows location map of the studied area.

### Soil sample collection, preparation and analysis

30 Soil samples were collected from the surface of the soil (0–30 cm) through systematic random sampling (4 × 4 km grid) and preserved by using the methods of soil analysis. Based on the information gathered through field trips and taking into account the type of land use and population concentrations, the number of samples extended by subdividing the grids in selected locations (2.2 km grid) (Figure 1). For each sampling point, four soil samples were collected and mixed properly to obtain a composite sample mixture. The soil sampling spots were cleared of debris prior to sampling. Each composite soil samples were placed in paper bags labeled then taken to the laboratory for pretreatment and analysis. The sampling tools, were washed with soap and rinsed with distilled water after each sampling [36]. In the laboratory, bulk soil samples were spread on trays and air dried at ambient conditions for two weeks. The samples were then grounded by mortar and pestle, sieved through a 2 mm mesh, and oven-dried at 50°C for about 48 hours. The samples were then stored in polyethylene bags and rehomogenized before being used. For analysis the soil samples were digested using the 11466 ISO standard methods (the aqua regia digestion method) [37] as 3 g of soil was placed in a 100 ml round bottom flask with 21 ml of concentrated HCl (35%) and 7 ml concentrated HNO_3_ (65%). The obtained solution was kept at room temperature overnight before a water condenser was attached and the solution heated to boiling point for 2 hours. Then 25 ml of water was added down the condenser before filtration of the mixture through using a Whatman (No. 42) filter. The filtered residue was rinsed twice with 5 ml of water and the solution was made up to 100 ml. The metals (Zn, Pb, Ni and Cd) in the soil extracts were analyzed by Inductively Coupled Plasma-Optical Emission Spectroscopy (ICP-OES, Spectro Gensis). All chemicals were high purity grade reagents from Merck or Fluka chemical companies.

### Leaf sample collection, preparation and analysis

Leaves of three native plant species namely; *Populus, Molus domestica* and *Salix alba* were collected from the studied village and compared with the leaves of the same trees from the vicinity of the NILZ company as well as from an unaffected location about 7 km away from the company (Mellat park east of Zanjan city). This was to see if the soil pollution by the studied heavy metals had positively affected the concentration of the metals in the studied plants.

Plant leaf (n = 18) samples were collected and analysed for their heavy metal content in August 2012. The leaf samples were stored separately in labelled polyethylene bags. Following cleaning and drying, leaf samples were accurately weighed into acid cleaned polypropylene tubes. 10 ml of aqua regia was added to each sample and the tubes were capped and left at room temperature. After 24 h, the digestive was heated to evaporate acids and diluted with 10 ml of deionised water, then filtered (0.4 μm) and diluted with HNO_3_ (1.0 molar). The filtered samples were analysed by ICP-OES for different heavy metals.

### Nail sample collection, preparation and analysis

Fingernail (n = 29) samples were collected in September 2012 from people living in the Dizajabaad village. For comparison, five fingernails were also collected from an unaffected area outside the province. Finger nail samples were collected using stainless steel nail clipper. The nail samples were stored separately in labelled polyethylene bags. The age range of the participants spanned between 9–65 years (median 35 years), and was skewed towards men (nails: 6 women, 23 men). Following cleaning, nail sample was accurately weighed into acid cleaned polypropylene tubes. Nails were then cleaned 5 times with water and acetone, and were dried at room temperature. 1 ml concentrated HNO_3_ (sub-distilled from Analytical grade 69% HNO_3_, BDH) was added to each sample and the tubes were capped and left at room temperature. After 48 h, the digestive was diluted with 9 ml of deionised water, then filtered (0.4 μm) into a fresh acid cleaned polypropylene tube. The filtered samples were analyzed by ICP-OES.

### Statistical analysis

SPSS (20.0 for Windows Evaluation Version) was used to perform statistical analyses of the data. Analysis of the experimental data was carried out by using Mann Whitney U and Kolmogorov-Smirnov. All significance statements reported in this study are at P < 0.05 level-of-significance. T-test and the Mann Whitney method, as parametric and nonparametric tests, respectively, allow testing the significant difference of the means between the two groups. The one-way analysis of variance (ANOVA method) and Kruskal-Wallis test allows testing the significant difference of the means. The latter is a non-parametric test without requirements announced for the ANOVA test. In this work both methods were tested for a comparison.

Parametric methods are known for their populations being approximately normal. In contrast, nonparametric methods are defined as the statistical techniques in which no assumption of normality is needed for the populations being studied. Normal distribution assumptions were checked by using Kolmogorov-Smirnov (K–S) test.

Principal Component Analysis (PCA), Factor Analysis (FA), Regression and Correlation analysis were used as statistical processes, for estimating the relationships among variables [33]. It helps to understand how the typical value of the dependent variable changes when any one of the independent variables varies, while others are held constant.

## Results and discussions

**Figure 2:**
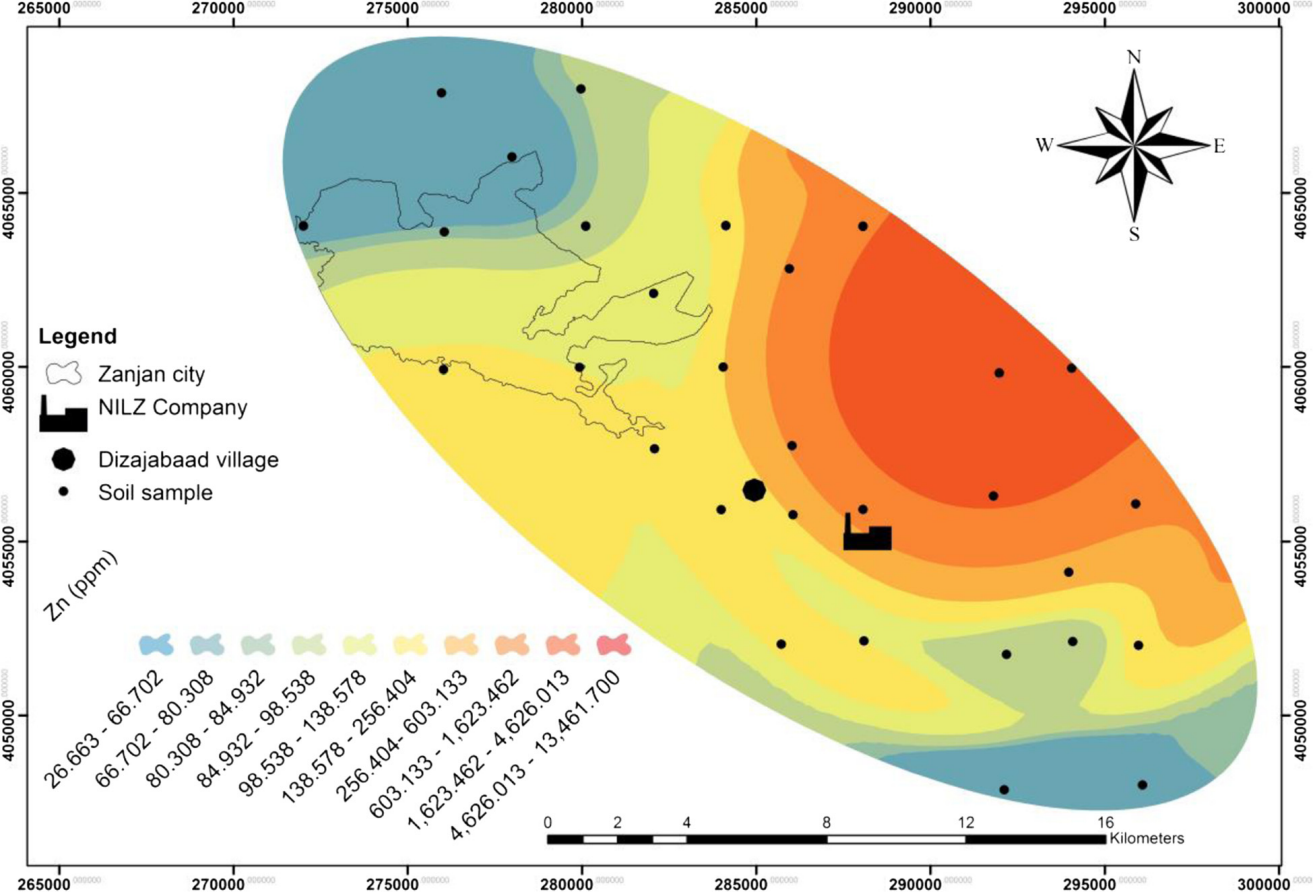
**Distribution of zinc in the surficial soils of the studied area.**

**Figure 3:**
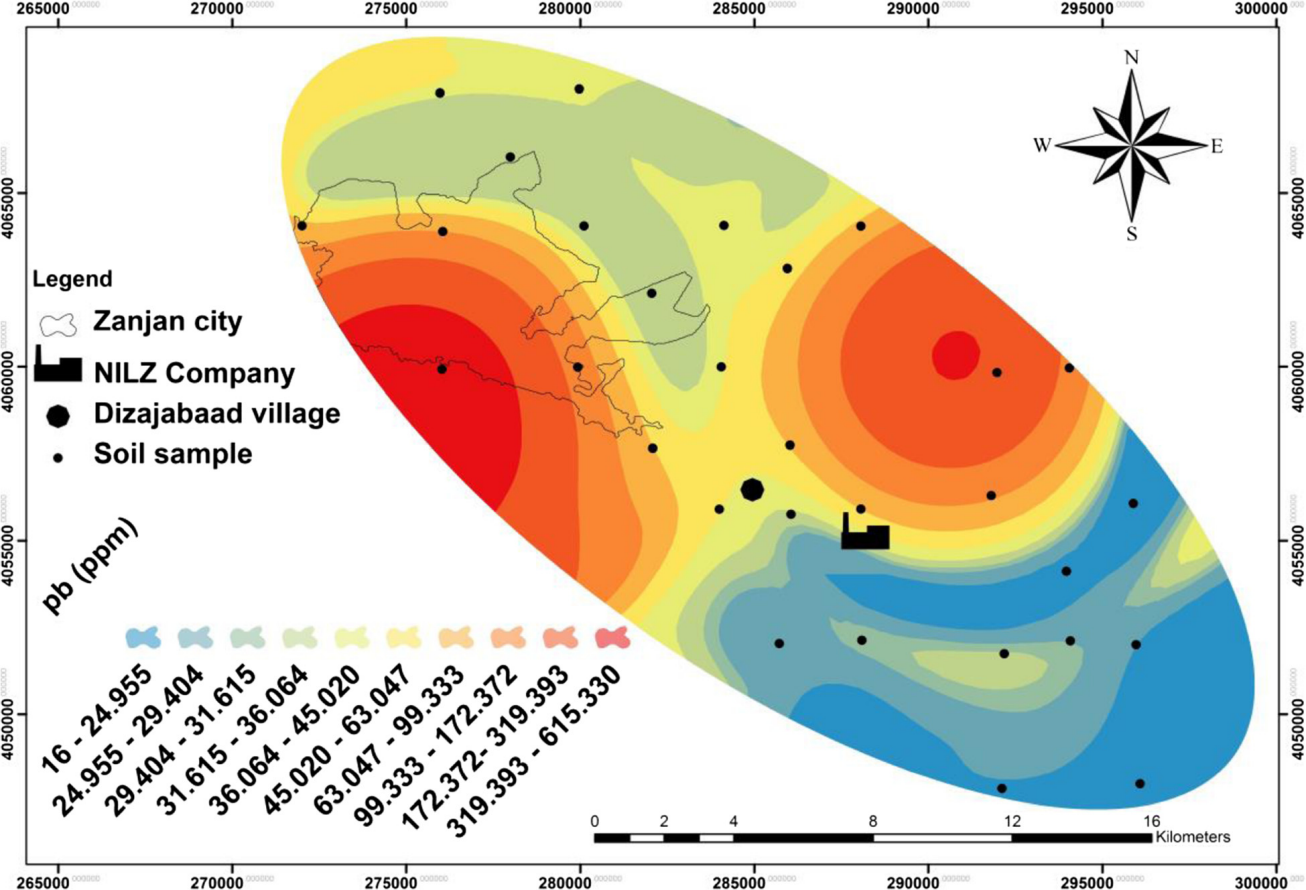
**Distribution of lead in the surficial soils of the studied area.**

**Figure 4:**
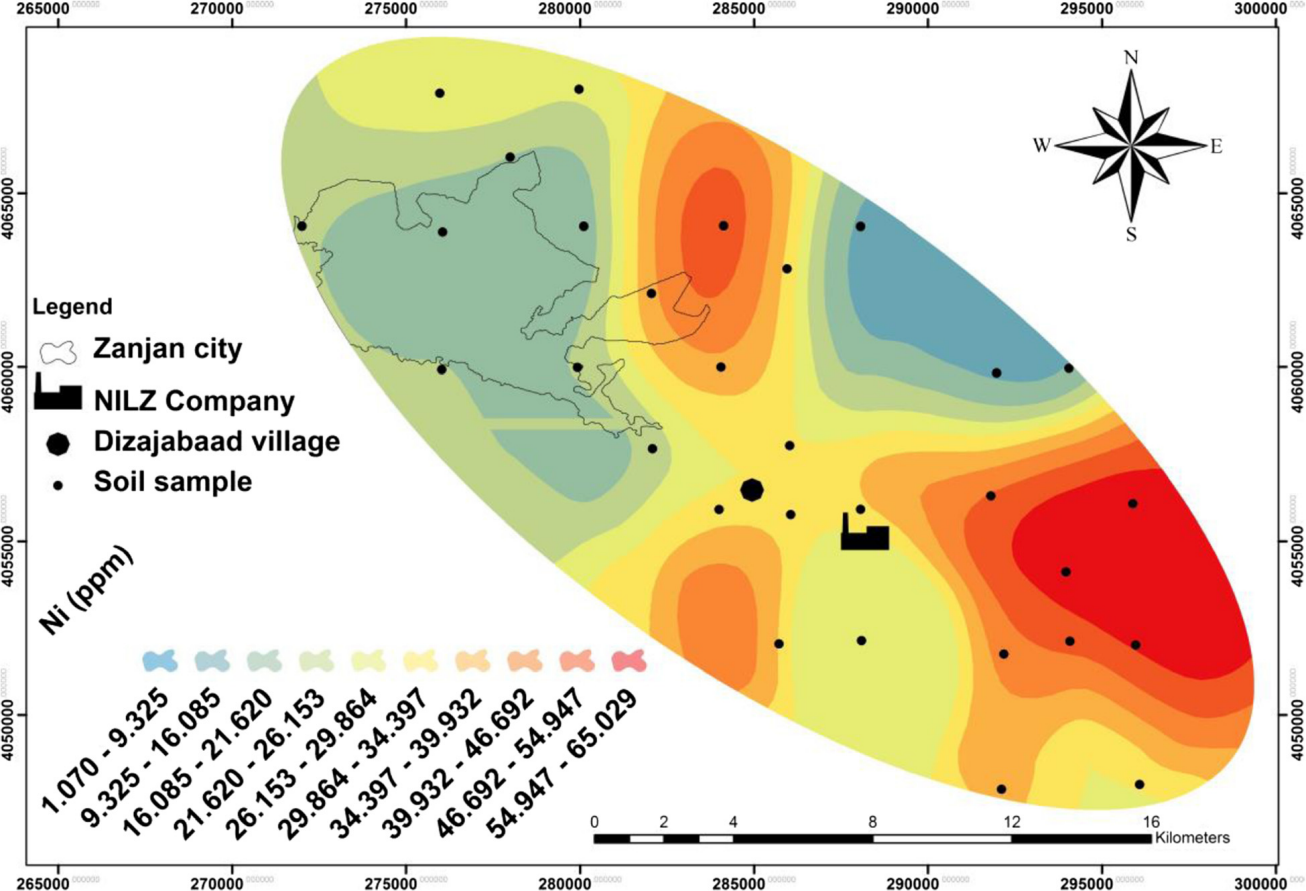
**Distribution of nickel in the surficial soils of the studied area.**

**Figure 5:**
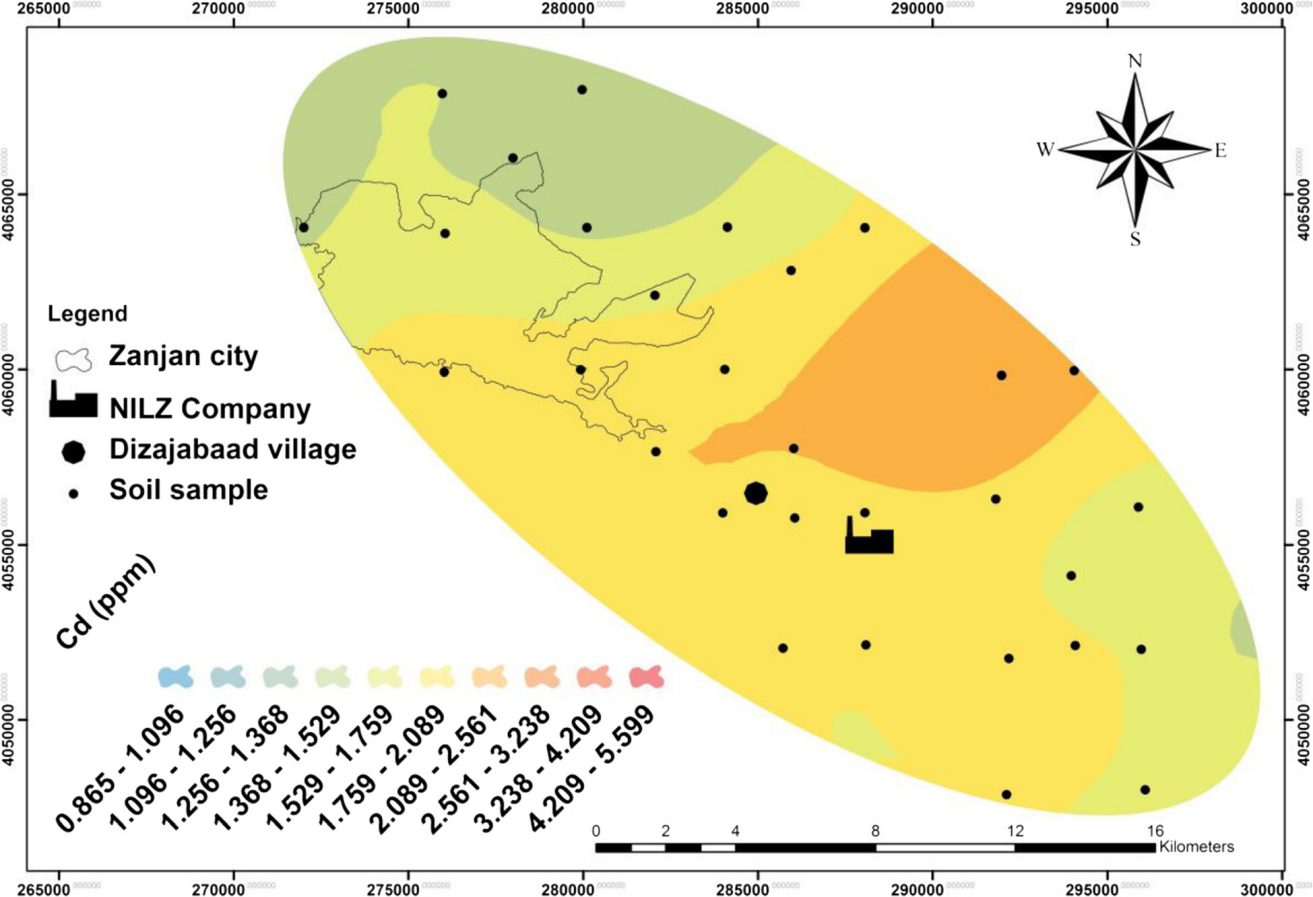
**Distribution of cadmium in the surficial soils of the studied area.**

Table 1 and Figures 2, 3, 4 and 5 show the concentration and distribution of each metal in soil samples within the studied area. Zinc concentration (Figure 2) is very high in Dizajabaad village located near NILZ Company and its damping site, indicating its anthropogenic source. Lead is also found in some localized areas with extremely high concentrations among samples of Dizajabaad village and NILZ damping area and can be related to this plant. High concentration of Pb in Zanjan city area may be attributed to the traffic and the use of leaded gasoline and also may be air borne (Figure 3). Nickel is also found in high concentration in surficial soils in south and south east of the area and its high concentration may be related to both agricultural fertilizer used in the cultivated lands in this component of the studied area as well as the NILZ company (Figure 4). For Cd the maximum concentration can be observed near the company (Figure 5).

**Table 1:** **Statistical description of metal concentrations in top soil (mg. kg** ^**−1**^ **)**

	**Pb**	**Zn**	**Cd**	**Ni**
N	30	30	30	30
Min.	16.00	26.66	0.87	1.07
Max.	615.33	13461.70	5.60	65.03
Variance	17339	5953797	0.81	241.91
Std. dev.	131.68	2440.04	0.90	15.55
Mean	72.60	546.47	1.80	32.13
USEPA	10	NR	-	40

The maximum concentration of all the studied metals were observed near the NILZ Company and the industrial areas within the area investigated. The average concentrations of Pb, Zn and Cd in sequence in top soils were 72.60, 546.47, 1.80 mg. kg^−1^ as against the average of the same metals according to the WHO standards 20, 50, 0.3 mg. kg^−1^ respectively. This means that the study area is polluted by these heavy metals. For Ni, the average concentration 32.13 mg. kg^−1^ is lower than the standard suggested by WHO (68 mg. kg^−1^).

Evaluation of the soil’s contamination levels were made by the assessment of Enrichment Factors (EF) and Geo-accumulation Index (I_geo_) [38,39]. These factors and indexes depend on the background metal levels of the samples. The average metal content in the world shale (World Average Shale, W.A.S.) and in the world soils are often used to provide background metal levels [38,39]. As noted before, the iron content of the soil samples are not related to anthropogenic activities, thus the mean Earth’s crust’s Fe concentration ratio was chosen as reference for determining EF and I_geo_ index. EF was calculated using equations 1.

1 $$ \mathrm{E}\mathrm{F}=\frac{{\left(\frac{\left[\mathrm{M}\right]}{\left[\mathrm{F}\mathrm{e}\right]}\right)}_{\mathrm{soil}}}{\kern1em {\left(\frac{\left[\mathrm{M}\right]}{\left[\mathrm{F}\mathrm{e}\right]}\right)}_{\mathrm{W}.\mathrm{A}.\mathrm{S}.}} $$

I_geo_ was obtained by equation 2:

2 $$ \mathrm{Igeo}= \log {}_2\left(\frac{{\left[\mathrm{M}\right]}_{\mathrm{soil}}}{1.5\ {\left[\mathrm{M}\right]}_{\mathrm{W}.\mathrm{A}.\mathrm{S}.}}\right) $$

where M is the investigated element, and the subscripts _“soil”_ and _“W.A.S”_ indicates the medium the concentration refers to.

Evaluated Enrichment Factors also show moderate to severe enrichment of the soil samples by Zn and Pb, and very strong enrichment by Cd.

**Figure 6:**
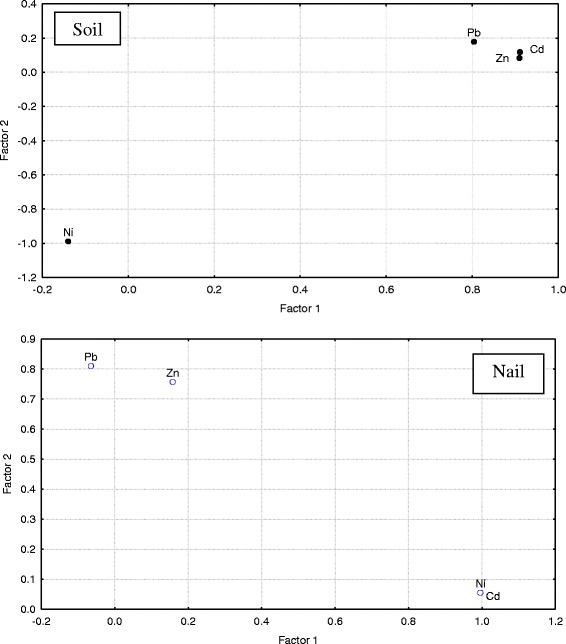
**Factor loading for studied heavy metals (Rotation: Varimax normalized; Extraction: Principal components).**

The major objective of factor analysis (FA) is to reduce the contribution of less significant variables to simplify even more the data structure given by PCA. This can be achieved by rotating the axis defined by PCA. This procedure allows constructing new variables (called factors). Factor loadings obtained by PCA with varimax for various metals are presented in Figure 6. The heavy metal grouping is shown in Figure 6 of the first two principal components generated from the main factors, Factors 1 and 2. Comparison of the results in nail and soil show that Pb, Zn and Ni have same source but entrance pathway of Cd in soil and nail is different. Recent studies on ground water samples using the same statistical methods also confirmed the anthropogenic source of the pollutants (Pb, Zn and Ni) in the studied area [33].

Average concentrations of heavy metals (Pb, Zn, Ni, Cd and As) in three different plant species (Populus, Molus domestica and Salix alba species) in Dizajabaad village, near NILZ Co, and in an unaffected area about 7 km away from the studied village are given in Table 2.

**Table 2:** **Average concentrations of some heavy metals in three different plant species in Dizajabaad village, NILZ Co, and in an unaffected area (mg. kg** ^**−1**^ **)**

**Sample locations**	**Distance from NILZ Co. (km)**	**As**	**Pb**	**Cd**	**Zn**	**Ni**
NILZ Company	0	15.92	135.55	2.642	260.83	1.10
Dizajabaad village	1	5.74	58.67	0.456	72.38	1.12
Avg. study area	0.5	10.83	97.11	1.55	166.61	1.11
An unaffected area*	7	0.74	21.49	0.001	11.83	0.88

*Mellat Park located in eastern periphery of Zanjan.

Monitoring of metal concentrations in leaf samples show a positive correlation with the concentration of metals in soil samples collected from the same area in which the highest concentrations are found near the NILZ Company and then decrease by increasing distance from it. High correlation coefficients were found for Zn (0.573) and Pb (0.584) with p < 0.05 and it was insignificant for Ni and Cd.

Linear relationship between the studied heavy metals in plant and soil show significant correlation between all the studied heavy metals except for Ni. Linear correlation coefficients (R^2^) were 0.452, 0.660 and 0.584 for Zn, Cd and Pb respectively.

In order to assess the relationship between heavy metal contents in soil and leaf samples, the sampled stations were divided into four distinct areas (Table 3). The results show a statistically significant difference (p < 0.05) depending on sampling locations. This also confirmed that by increasing heavy metal content in soil samples, the amount of its uptake by studied plants is raising.

**Table 3:** **Classification of studied area considering the concentrations of some heavy metals in soil samples (mg heavy metals per kg soil)**

**Sample locations**	**Pb**	**Zn**	**Cd**	**Ni**
Area 1	16.00- 30.00	26.00-80.00	0.86-1.26	1.07-16.08
Area 2	30.00-45.00	80.00-138.00	1.26-1.76	16.08-29.86
Area 3	45.00-99.00	138.00-603.00	1.76-2.56	29.86-39.93
Area 4	99.00-615.00	603.00-13461.00	2.56-5.60	39.93-65.03

Metal concentrations in fingernail samples collected from Dizajabaad village and from an unaffected area are given in Table 4 in mg. kg^−1^. It was observed that the average Pb, Zn, Cd, Ni and As in sequence in nail samples from Dizajabaad village are: 15.15, 68.46, 1.18, 18.23, 15.48 mg. kg^−1^ as against the average of these metals in finger nail samples from an unaffected area 2.80, 29.20, 0.00, 4.60, 0.74 mg. kg^−1^ respectively. This means that the people living in the Dizajabad village are severely affected by these heavy metals. To the best of our knowledge, no human nail certified reference material is available for comparison. The absence of well-defined reference concentration ranges is also mentioned in the literature (as for example; [40-42]).

**Table 4:** **Metal concentrations in fingernail samples collected from people living in Dizajabaad village and from an unaffected area (mg. kg** ^**−1**^ **)**

**Location**		**Pb**	**Zn**	**Cd**	**Ni**	**As**
Dizajabaad Village	N	29	29	29	29	29
	Minimum	0.0	32.0	0.0	0.0	0.0
	Maximum	123.0	176.0	11.0	79.0	50.0
	Variance	547.14	886.63	6.97	211.88	237.28
	Mean	15.16	68.46	1.18	18.23	15.48
	Std. Dev.	23.39	29.78	2.64	14.55	15.40
Outside Zanjan province (Unaffected area)	N	5	5	5	5	5
	Minimum	0.0	3.0	0.0	3.0	0.0
	Maximum	8.0	46.0	0.0	7.0	3.0
	Variance	15.20	262.20	0.00	2.80	1.68
	Mean	2.80	29.20	0.00	4.60	0.74
	Std. Dev.	3.89	16.19	0.00	1.67	1.29

## Conclusion

Soil samples collected from the studied area showed that the Dizajabaad village and its surrounding areas are polluted by heavy metals notably with lead, zinc and cadmium. Evaluated Enrichment Factors shows moderate to severe enrichment of the soil samples by Zn and Pb, and very strong enrichment by Cd. Principal Component Analysis and Factor Analysis also elucidate the anthropogenic source of the pollutants. Leaves of three native plant species namely; *Populus, Molus domestica and Salix alba* collected from the studied village compared with the leaves of the same trees from an unaffected location demonstrate that the soil pollution by the studied heavy metals had positively affected the concentration of the metals in the studied plant species. Analysis for different heavy metal contents of nail samples collected from people living in the studied area, compared with samples from an unaffected area, reveals very high levels of the same toxic elements found in soil/plant samples as well as the available values indicated in the literature. Furthermore, there were close associations established between the specific metals in soil, plants and nails. This means that the people living in the Dizajabad village are severely affected by these heavy metals and anthropogenic sources; notably NILZ Company is mostly responsible for the high concentration of these metals in soil, plants and biological tissue samples. The present study demonstrated that determination of metals in finger nails of the residence has potential utility as a biomarker to heavy metals from the environmental exposure. An overall view of the results obtained for toxic heavy metals establish the use of nails as a biomarker and diagnostic tool in medical sciences. More number of researches on a larger number of populations will enhance the merits of such investigations and their application in medical sciences.
